# Effects of asymmetric dimethylarginine on bovine retinal capillary endothelial cell proliferation, reactive oxygen species production, permeability, intercellular adhesion molecule-1, and occludin expression

**Published:** 2011-02-01

**Authors:** Yi-Hui Chen, Xun Xu, Min-Jie Sheng, Zhi Zheng, Qing Gu

**Affiliations:** 1 Department of Ophthalmology, Shanghai First People’s Hospital, Shanghai Jiaotong University, Shanghai, China; 2 Department of Ophthalmology, Shanghai Tenth People’s Hospital, Tongji University, Shanghai, China

## Abstract

**Purpose:**

Asymmetric dimethylarginine (ADMA), an endogenous competitive inhibitor of nitric oxide synthase, is associated with impaired endothelial dysfunction, such as chronic heart failure, hypertension, diabetes, and pulmonary hypertension. The effects of ADMA on cell proliferation, reactive oxygen species (ROS) production, cell permeability, intercellular adhesion molecule-1 (ICAM-1), and tight-junction protein occludin levels in bovine retinal capillary endothelial cells (BRCECs) were investigated.

**Methods:**

A cell proliferation assay was performed using the novel tetrazolium compound 3-(4,5-dimethylthiazol-2-yl)-5-(3-carboxymethoxyphenyl)-2-(4-sulfophenyl)-2H-tetrazolium and an electron coupling reagent. Intracellular ROS levels were determined using the fluorescent probe CM-H_2_DCFDA. Horseradish peroxidase was used for a permeability assay. ICAM-1 and tight-junction protein occludin were assessed by western blotting and quantitative real-time PCR.

**Results:**

Cell proliferation was significantly inhibited by ADMA. ADMA increased intracellular ROS generation in BRCECs. The increased ROS production induced by ADMA was markedly inhibited by the angiotensin II receptor-blocker telmisartan, the angiotensin-converting enzyme inhibitor benazepril, the reduced form of nicotinamide-adenine dinucleotide phosphate (NADPH) oxidase inhibitor diphenyliodonium (DPI), or the antioxidant and free-radical scavenger N-acetyl-l-cysteine (NAC). ADMA significantly increased horseradish peroxidase (HRP) permeability in BRCECs. Benazepril, telmisartan, DPI, and NAC downregulated cell permeability. ADMA markedly upregulated ICAM-1 expression in BRCECs, which were downregulated by telmisartan, DPI, and NAC. ADMA significantly downregulated occludin expression in BRCECs. Benazepril and telmisartan upregulated occludin expression in BRCECs exposed to ADMA.

**Conclusions:**

Our results provide the first reported evidence that ADMA has potent adverse effects on cell proliferation, intracellular ROS generation, cell permeability, levels of ICAM-1, and the tight-junction protein occludin. Angiotensin-converting enzyme inhibitors, angiotensin II receptor blockers, and antioxidants are effective inhibitors of the adverse effects of ADMA.

## Introduction

Asymmetric dimethylarginine (ADMA), an endogenous competitive inhibitor of nitric oxide synthase, is generated in the presence of type 1 protein arginine N-methyltransferase (PRMT-1) and is metabolized by dimethylarginine dimethylaminohydrolases (DDAHs) [1]. Elevated ADMA concentration in plasma is associated with impaired endothelial dysfunction, such as in chronic heart failure, hypertension, renal failure, diabetes, and pulmonary hypertension [2-4]. ADMA is also related to endothelial dysfunction in diabetic complications. Our previous studies suggested that PRMT-1- and DDAH-induced ADMA upregulation was involved in reactive oxygen species (ROS)- and renin-angiotensin system (RAS)-mediated diabetic retinopathy (DR), which may be a novel mechanism for the development or progression of DR [5]. Angiotensin-converting enzyme inhibitor (ACEI), angiotensin II receptor blocker (ARB), or antioxidants can be used to reduce ROS production and lower ADMA concentrations, thus ameliorating endothelial dysfunction and improving prognosis in DR [5].

DR is a leading cause of acquired visual impairment in working-age adults in developed countries [6]. The precise mechanism underlying the progression of DR remains unclear. Several biochemical abnormalities, such as excessive nonenzymatic glycation [7], activation of the aldose reductase pathway [8], activation of protein kinase C [9], and oxidative stress [10], have been identified as being involved in the pathogenesis of DR. Oxidative stress induced by hyperglycemia is thought to play a significant role in DR and to contribute to endothelial dysfunction [11]. Increases in ROS level are correlated with increased leukocyte adhesion to the retinal vasculature (leukostasis) and breakdown of the blood-retinal barrier (BRB) [12,13]. Breakdown of the BRB and leukostasis are hallmarks of DR. Increased leukostasis in the early stages of DR occurs through the upregulation of intercellular adhesion molecule-1 (ICAM-1) [14]. Diabetes-induced BRB breakdown is associated with reduced expression of the tight-junction protein occludin, and with its redistribution within the retinal vascular endothelium [15].

Recent studies indicated that ADMA regulates endothelial permeability and endothelial barrier function [16]. The present study was performed to investigate whether ADMA affects cell proliferation, ROS production, cell permeability, ICAM-1, and tight-junction protein occludin expression in bovine retinal capillary endothelial cells (BRCECs). Moreover, we observed the interfering effects of ACEI, ARB, and antioxidants on the above changes, to assess the role of ADMA in retinal capillary endothelial permeability and endothelial barrier function.

## Methods

### Cell culture

BRCECs were cultured as described previously [17]. Briefly, BRCECs were cultured in endothelial cell medium (ECM; ScienCell Research Labs, Carlsbad, CA) consisting of 5% fetal bovine serum, 1% endothelial cell growth supplement, and 1% penicillin/streptomycin solution. Endothelial cells at passage 3–5 were used in the following experiments, including the cell proliferation assay, examination of ROS levels, permeability assay, western blotting analysis, and quantitative real-time (RT)-PCR. The cells were washed when at 80% confluence and were cultured overnight with endothelial cell basal medium, consisting of 0.4% fetal bovine serum and 1% penicillin/streptomycin solution. The cells were then incubated with 100 μM ADMA (Sigma, St. Louis, MO) and 100 μM ADMA plus 10 μM benazepril (Sigma), 10 μM telmisartan (Sigma), 10 μM diphenyliodonium (DPI, an reduced form of nicotinamide-adenine dinucleotide phosphate [NADPH] oxidase inhibitor; Sigma), or 10 mM N-acetyl-l-cysteine (NAC, an antioxidant and free radical scavenger; Sigma). The control group was cultured in endothelial cell basal medium, consisting of 0.4% fetal bovine serum and 1% penicillin/streptomycin solution. Cells were harvested after 24 h for western blotting analysis, quantitative RT–PCR analysis, and examination of ROS levels.

### Cell proliferation assay

Cell proliferation assay was performed using the novel tetrazolium compound, 3-(4, 5-dimethylthiazol-2-yl)-5-(3-carboxymethoxyphenyl)-2-(4-sulfophenyl)-2H-tetrazolium (MTS), and the electron-coupling reagent, phenazine ethosulfate (PES). PES has enhanced chemical stability, which allows it to be combined with MTS to form a stable solution. This convenient “one solution” format is an improvement over the traditional method, where phenazine methosulfate (PMS) is used as the electron-coupling reagent, and PMS solution and MTS solution are supplied separately. The MTS tetrazolium compound (Owen’s reagent) is bioreduced by cells into a colored formazan product that is soluble in tissue culture medium. This conversion is presumably accomplished by NADPH or reduced form of nicotinamide-adenine dinucleotid (NADH), produced by dehydrogenase enzymes in metabolically active cells [18]. The quantity of formazan product, as determined by measuring the absorbance at 490 nm, is directly proportional to the number of living cells in the culture. Endothelial cells were plated in 96-well culture plates at an optimal density of 1×10^5^ cells/ml with 100 μl of culture medium per well. After 3 days, cells were cultured with endothelial cell basal medium, consisting of 0.4% fetal bovine serum and 1% penicillin/streptomycin solution, overnight. The cells were then incubated with 10 μM, 50 μM, 100 μM, and 200 μM ADMA for 24–72 h. Then, 20 μl of CellTiter 96^®^ AQueous One Solution Reagent (Promega, Madison, WI) were pipetted into each well of the 96-well assay plates containing the samples in 100 μl of culture medium. The plates were incubated at 37 °C for 1–4 h in a humidified, 5% CO_2_ atmosphere. The optical density of each sample was determined immediately on an enzyme linked immunosorbent assay (ELISA) microplate reader (Wallac 1420; PerkinElmer, Waltham, MA) at 490 nm. Samples were tested in duplicate. The corrected absorbance at 490 nm (y-axis) was plotted against the incubation time of cells with ADMA (x-axis).

### Examination of reactive oxygen species levels in bovine retinal capillary endothelial cells

Intracellular ROS levels in BRCECs were determined using CM-H_2_DCFDA (Invitrogen, Carlsbad, CA). Confluent BRCECs in 6-well plates were collected, centrifuged, washed with phosphate-buffered saline (PBS), which contained 1.06 mM monobasic potassium phosphate, 155.17 mM sodium chloride, and 2.97 mM dibasic sodium phosphate and incubated with 10 μM CM-H_2_DCFDA at 37 °C for 30 min. BRCECs incubated with PBS and dimethyl sulfoxide served as negative controls. The levels of fluorescence were immediately determined by flow cytometry (XL-4; Beckman-Coulter, Fullerton, CA).

### Permeability assay

BRCECs (1×10^5^ cells/ml) were plated in double-chamber tissue culture plates (Transwell, 24-well filter chambers with 0.4 μm pore size membrane; Costar, Coring Inc., New York, NY). At 80% confluence, cells were cultured with endothelial cell basal medium, consisting of 0.4% fetal bovine serum and 1% penicillin/streptomycin solution, overnight. The cells were then incubated with 100 μM ADMA, and 100 μM ADMA plus benazepril (10 μM), telmisartan (10 μM), DPI (10 μM), or NAC (10 mM) for 24 h. Controls were cultured with endothelial cell basal medium, consisting of 0.4% fetal bovine serum and 1% penicillin/streptomycin solution.

For the permeability assay, horseradish peroxidase (HRP, 40 kDa; Sigma) was added to the upper chambers at a final concentration of 50 μg/ml. Aliquots of 5 μl were collected from the lower chamber after 15 min, 30 min, 45 min, and 1 h. The concentrations of HRP were determined in 5 μl aliquots added to 195 μl of freshly made substrate (o-phenylenediamine, 400 μg/ml in 0.05 mM citric acid and 0.1 mM phosphate, with 0.012% hydrogen peroxidase, pH 5.0). The reaction was terminated by the addition of 50 μl of 0.3 mM sulfuric acid after 15 min, and optical density was determined using a microplate reader (PerkinElmer, Boston, MA) at 490 nm. A standard curve was prepared from HRP serial dilutions in each experiment, and the samples were diluted such that all readings fell within the linear range of the standard curve. The readings for each tracer were then converted to nanograms per milliliter by comparison with standard curves generated using tracer samples taken at time zero. Permeability was calculated as flux: (ml/cm^2^)=(X)B/[(Y)i*A], where (X)B (μg) is the level of HRP in the lower chamber, (Y)i (μg/ml) is the concentration of HRP in the upper chamber, and A (cm^2^) is the effective surface area of the insert. Each experiment was repeated at least three times.

### Western blotting analysis of intercellular adhesion molecule-1 and occludin expression

Cells were sonicated in Tris-buffered saline (TBS) containing protease inhibitors. After sonication, the lysate was centrifuged (12,000× g, 15 min, 4 °C) and the supernatant was transferred to a fresh tube. The protein content was quantified with a Pierce protein assay kit (Pierce, Rockford, IL). Samples of equal concentration (80 μg/lane) were separated on 10% sodium dodecyl sulfate polyacrylamide gel electropheresis (SDS–PAGE) and transferred onto polyvinylidene difluoride (PVDF) transfer membranes (Immobilon P; Millipore, Billerica, MA). The membranes were blocked in TBS containing 0.1% Tween-20 and 5% nonfat dry milk for 2 h, followed by overnight incubation at 4 °C with polyclonal antibodies for ICAM-1 (Abcam, Cambridge, UK) or occludin (Invitrogen, Carlsbad, CA) at 1:1,000 dilution. After rinsing in TBS with Tween-20 (TBST), the membranes were incubated for 2 h with an HRP-conjugated secondary antibody against mouse IgG (Dako, Glostrup, Denmark) in a 1:1,000 dilution and rinsed with TBST, and bands on the blots were then detected using SuperSignal West Pico Chemiluminescent Substrate (Pierce). The densities of the bands were analyzed using Gel-Pro Analyzer (Media Cybernetics, Bethesda, MD). The expression of β-actin (1:5000; monoclonal anti-β-actin; Sigma) was used as an internal control.

### Quantitative real-time PCR

After removal of the culture medium, cells were washed with PBS, then combined with the TRIzol reagent (Invitrogen). Extracted RNA was then quantified spectrophotometrically at 260 nm and integrity was assessed by agarose-formaldehyde gel electrophoresis. Total RNA samples were treated with DNase I (RQ1; Promega) and then reverse transcribed using a ReverTra Ace RT–PCR kit (Toyobo, Osaka, Japan) according to the manufacturer’s instructions. Primers were designed using DNA Star software according to the guidelines supplied with the software. The primer sequences were described in Table 1.

**Table 1:** Primer sequences for quantitative RT–PCR.

**Gene**	**Probe (5′-3′)**	**Forward primer (5′-3′)**	**Reverse primer (5′-3′)**
ICAM-1	CCACGGAGCAGCACCACGGT	GTGACCAGCCCAAGTTGT	TCCCGTTTCAGCTCCTTCT
occludin	AAACCGCTTGTCATTCACTTTGCCA	GGGACAAGGAACACATTTATGAT	TGGATTTATAGGAAGACTCTGGAT
18S rRNA	CGCCTGCTGCCTTCCTTGGATGTG	AGTCGCCGTGCCTACCAT	CGGGTCGGGAGTGGGTAAT

To exclude DNA interference, primers were designed to span at least one intron. To quantify the amounts of specific mRNA (mRNA) in the samples, we generated a standard curve for each run using a plasmid (pGEM-T Easy Vector; Promega) containing the gene of interest as a standard. This enabled standardization of the initial mRNA content of cells relative to the amount of 18S rRNA. PCR assays were performed using SLAN® RT–PCR system (Hongshi, Shanghai, China). The quantitative RT–PCR solution consisted of 2.0 μl of diluted RT–PCR product, 0.5 μl of each primer pair, 25 μl of RT–PCR Master Mix (Toyobo, Osaka, Japan), 1.0 μl of fluorogenic probe, and 10 μl of PCR-grade water. The amplification conditions for ICAM-1 and occluding were as follows: 94 °C for 3 min, followed by 40 cycles of 94 °C for 20 s, 55 °C for 20 s, and 72 °C for 20 s. The results of quantitative RT–PCR were analyzed using the relative standard curve method with the SLAN software (v. 5.0). Values were normalized relative to the relative amounts of 18S rRNA, which were obtained from a similar standard curve.

### Statistical analysis

All results are expressed as means±standard deviation unless otherwise indicated. Statistical evaluation was performed with the SPSS software (ver. 14.0 for Windows; SPSS, Chicago, IL) using ANOVA with multiple comparisons between groups and Pearson’s correlation test. In all analyses, p<0.05 was taken to indicate statistical significance.

## Results

### Cell proliferation assay

**Figure 1:**
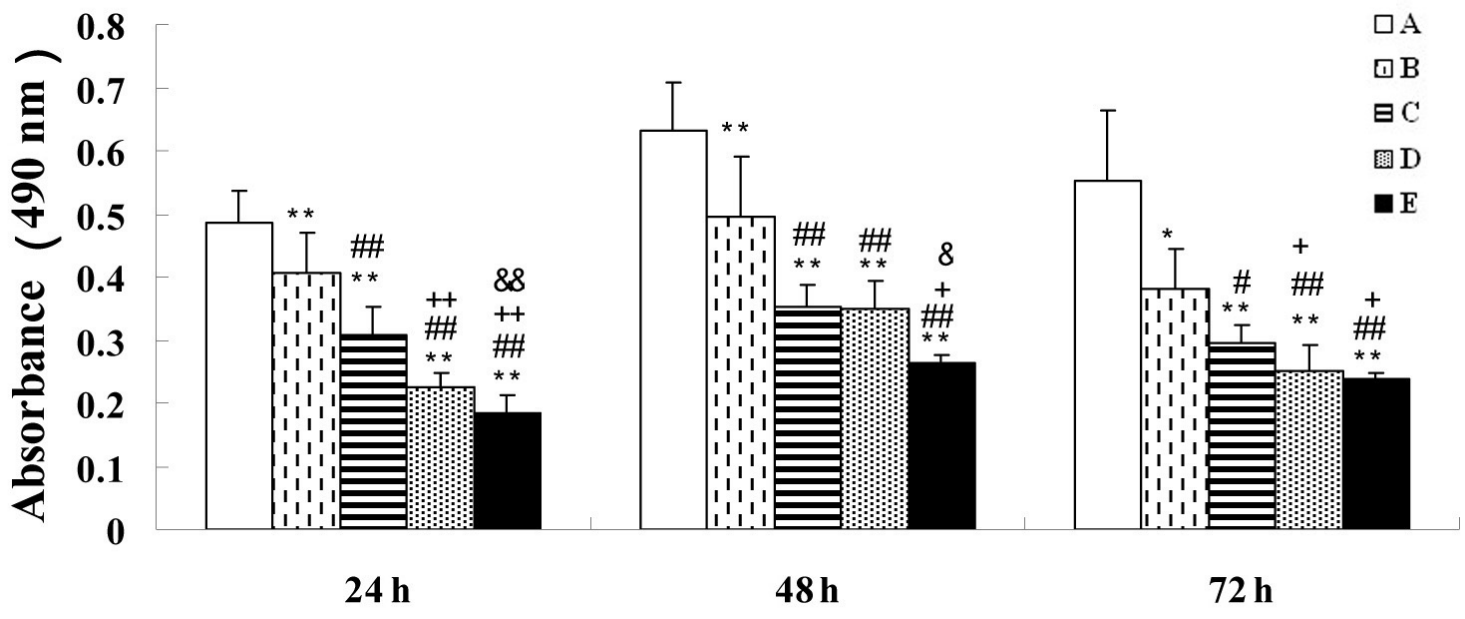
Cell proliferation analysis of bovine retinal capillary endothelial cells. Cell proliferation was significantly inhibited by asymmetric dimethylarginine (ADMA) for 24–72 h. In group A, bovine retinal capillary endothelial cells (BRCECs) were cultured in endothelial cell medium (ECM) with 0.4% fetal bovine serum (FBS). In groups B throught E, BRCECs were cultured in ECM with 0.4% FBS plus 10 μM, 50 μM, 100 μM, or 200 μM ADMA. Results are expressed as absorbance at 490 nm and represent means±SEM, n=8: *p<0.05 versus group A, **p<0.01 versus group A, #p<0.05 versus group B, ##p<0.01 versus group B, +p<0.05 versus group C, ++p<0.01 versus group C, &p<0.05 versus group D, &&p<0.01 versus group D.

Cell proliferation was significantly inhibited after incubation of BRCECs with 10 μM, 50 μM, 100 μM, or 200 μM ADMA for 24–72 h. Moreover, cell proliferation showed more significant inhibition with increasing ADMA concentration (Figure 1).

### Reactive oxygen species determination

**Figure 2:**
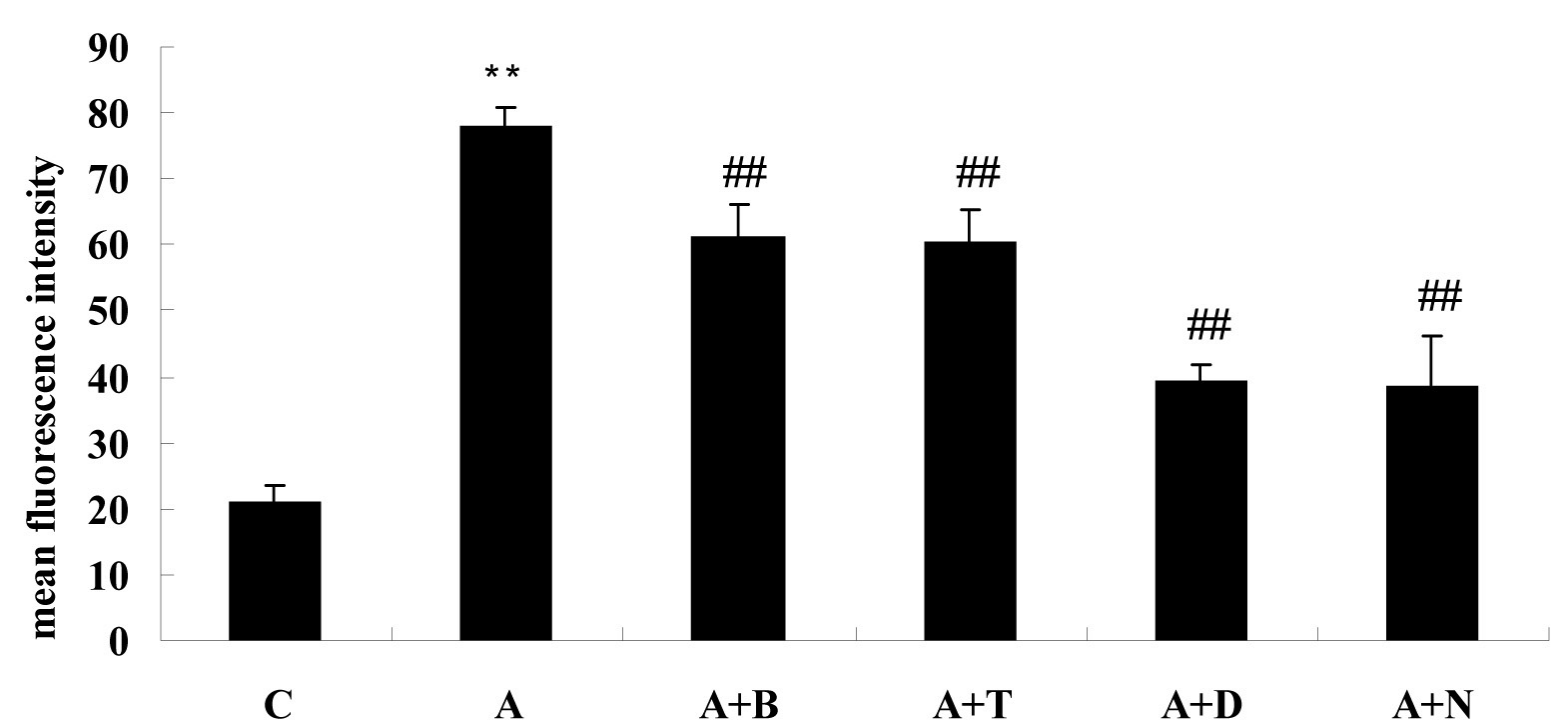
Intracellular reactive oxygen species generation in bovine retinal capillary endothelial cells for 24 h, as determined using the fluorescent probe CM-H_2_DCFDA. symmetric dimethylarginine (ADMA) increased intracellular reactive oxygen species (ROS) generation in bovine retinal capillary endothelial cells (BRCECs). The increased reactive oxygen species (ROS) production induced by asymmetric dimethylarginine (ADMA) was markedly inhibited by benazepril, telmisartan, diphenyliodonium, or N-acetyl-l-cysteine. In group C, BRCECs were cultured in endothelial cell medium with 0.4% fetal bovine serum. In group A, BRCECs were cultured in endothelial cell medium with 0.4% fetal bovine serum plus ADMA (100 μM). In group “A+B,” BRCECs were cultured in the same media as A and 10 μM benazepril. In group “A+T,” BRCECs were cultured in the same media as A and 10 μM telmisartan. In group “A+D,” BRCECs were cultured in the same media as A and 10 μM diphenyliodonium. In group “A+N,” BRCECs were cultured in the same media as group A and 10 mM N-acetyl-l-cysteine (mean±SD, n=3). **p<0.01 versus group C, ##p<0.01 versus group A.

After incubation with ADMA (100 μM) for 24 h, intracellular ROS generation in BRCECs was significantly increased, compared with those incubated in normal medium (p<0.01). The increased ROS production induced by ADMA was markedly inhibited by benazepril (10 μM), telmisartan (10 μM), DPI (10 μM), or NAC (10 mM; all p<0.01; Figure 2).

### Permeability assay

**Figure 3:**
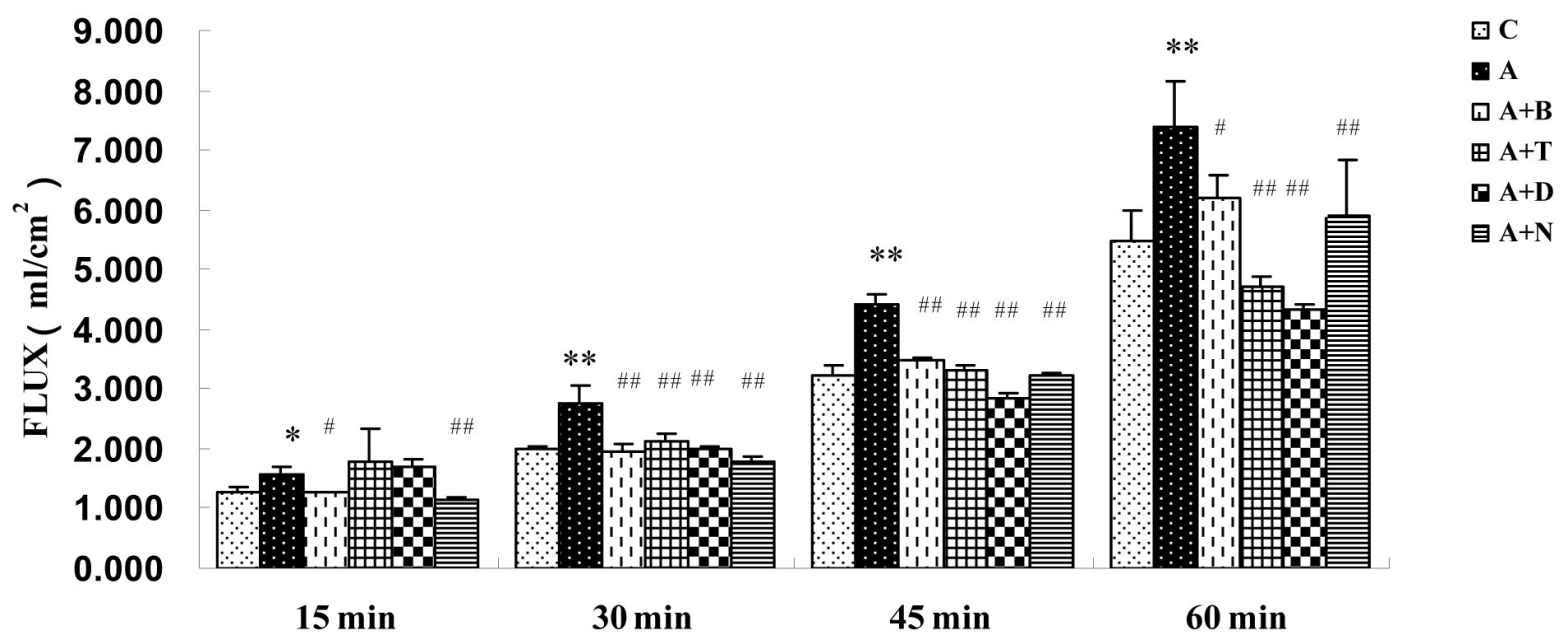
Horseradish peroxidase (HRP) permeability assay of bovine retinal capillary endothelial cells (BRCECs). Asymmetric dimethylarginine (ADMA) significantly increased HRP permeability in BRCECs. Treatment with benazepril and NAC for 15 min decreased HRP permeability in BRCECs. The permeability increase by ADMA was significantly downregulated by benazepril, telmisartan, diphenyliodonium (DPI), or N-acetyl-l-cysteine (NAC) for 30 min, 45 min, and 1 h. The group C bars are that BRCECs were cultured in endothelial cell medium (ECM) with 0.4% FBS. In group A, BRCECs were cultured in ECM with 0.4% FBS plus ADMA (100 μM). In group “A+B,” BRCECs were cultured in the same media as A and 10 μM benazepril. Group “A+T” was the same as for group A and 10 μM telmisartan. In group “A+D,” BRCECs were cultured in the same media as group A and 10 μM DPI. In group “A+N,” BRCECs were cultured in the same media as group A and 10 mM NAC (mean±SD, n=3). *p<0.05 versus group C, **p<0.01 versus group C, #p<0.05 versus group A, ##p<0.01 versus group A.

ADMA (100 μM) significantly increased HRP permeability in BRCECs. Treatment with benazepril (10 μM) and NAC (10 mM) for 15 min decreased HRP permeability in BRCECs exposed to ADMA (100 μM). The increase in permeability by ADMA (100 μM) was significantly downregulated by benazepril (10 μM), telmisartan (10 μM), DPI (10 μM), or NAC (10 mM) for 30 min, 45 min, and 1 h (Figure 3).

### Western blotting analysis

**Figure 4:**
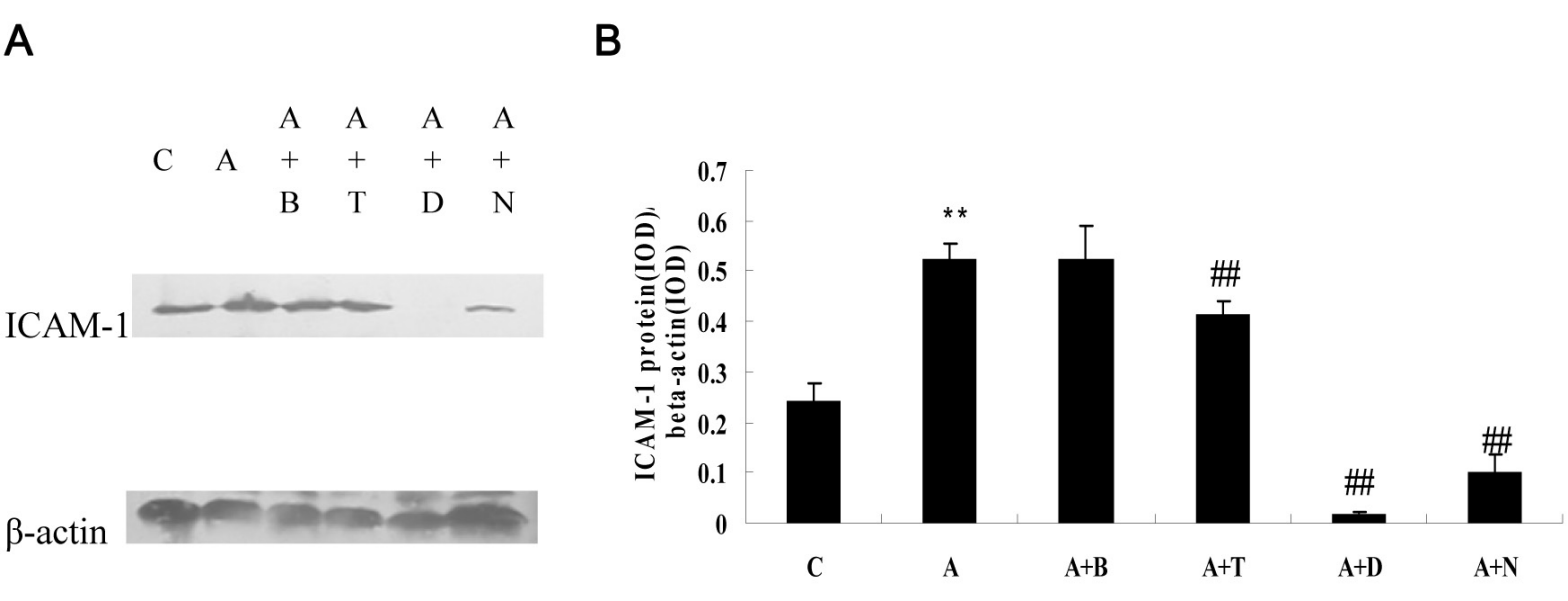
Intercellular adhesion molecule-1 (ICAM-1) protein expression in bovine retinal capillary endothelial cells. **A**: western blotting analysis showing the presence of ICAM-1 protein in bovine retinal capillary endothelial cells (BRCECs). **B**: Results of statistical analysis of protein levels relative to β-actin. Asymmetric dimethylarginine (ADMA) markedly upregulated ICAM-1 protein expression in BRCECs. Telmisartan, diphenyliodonium (DPI), or N-acetyl-l-cysteine (NAC) downregulated ICAM-1 protein expression in BRCECs exposed to ADMA. Benazepril had no effect on ICAM-1 expression. In group C, BRCECs wrre cultured in endothelial cell medium (ECM) with 0.4% fetal bovine serum (FBS). In group A, BRCECs were cultured in ECM with 0.4% FBS plus ADMA (100 μM). In group “A+B,” BRCECs were cultured in the same media as A and 10 μM benazepril. In group “A+T,” BRCECs were cultured in the same media as group A and 10 μM telmisartan. In group “A+D,” BRCECs were cultured in the same media as group A and 10 μM DPI. In group “A+N,” BRCECs were cultured in the same media as A and 10 mM NAC (mean±SD, n=3). **p<0.01 versus C, ##p<0.01 versus group A.

Western blotting analysis indicated that exposure to ADMA (100 μM) for 24 h markedly upregulated ICAM-1 protein expression in BRCECs (p<0.01). Treatment with telmisartan (10 μM), DPI (10 μM), or NAC (10 mM) downregulated ICAM-1 protein expression in BRCECs exposed to ADMA (100 μM; p<0.01). Benazepril (10 μM) had no effect on ICAM-1 expression (p>0.05; Figure 4).

**Figure 5:**
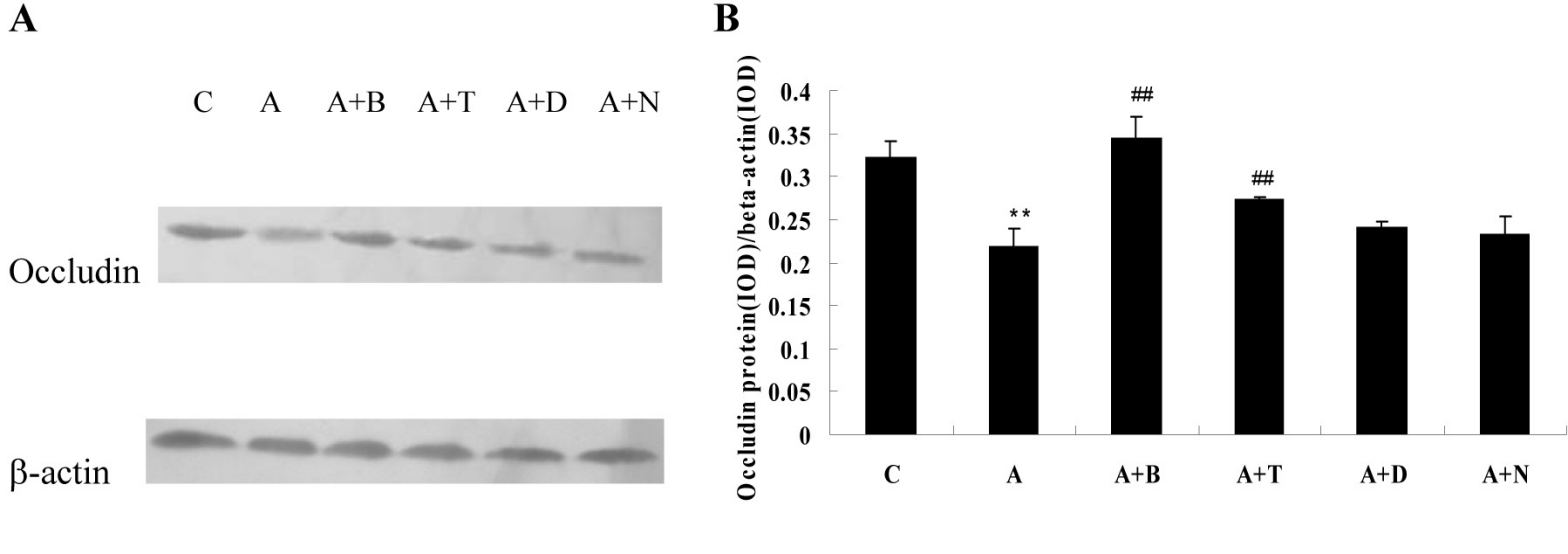
Occludin protein expression in bovine retinal capillary endothelial cells. **A**: western blotting analysis showing the presence of occludin protein in bovine retinal capillary endothelial cells (BRCECs). **B**: The results of statistical analysis of protein level relative to β-actin. Asymmetric dimethylarginine (ADMA) significantly downregulated occludin protein expression in BRCECs. Benazepril and telmisartan upregulated protein expression of occludin in BRCECs exposed to ADMA. However, diphenyliodonium (DPI) and N-acetyl-l-cysteine (NAC) had no effect on occludin expression. In group C, BRCECs were cultured in ECM with 0.4% fetal bovine serum (FBS). In group A, BRCECs were cultured in ECM with 0.4% FBS plus ADMA (100 μM). Group “A+B” represents same as for group A and 10 μM benazepril. Group “A+T” represents same as for group A and 10 μM telmisartan. Group “A+D” represents same as for group A and 10 μM DPI. Group “A+N” represents same as for group A and 10 mM NAC (mean±SD, n=3). **p<0.01 versus group C, ##p<0.01 versus group A.

Experiments performed in vitro also showed that exposure to ADMA (100 μM) for 24 h significantly downregulated occludin protein expression in BRCECs (p<0.01). Treatment with benazepril (10 μM) or telmisartan (10 μM) upregulated occludin protein expression in BRCECs exposed to ADMA (p<0.01), while DPI (10 μM) and NAC (10 mM) had no effect on occludin expression (p>0.05; Figure 5).

### Quantitative real-time PCR analysis

**Figure 6:**
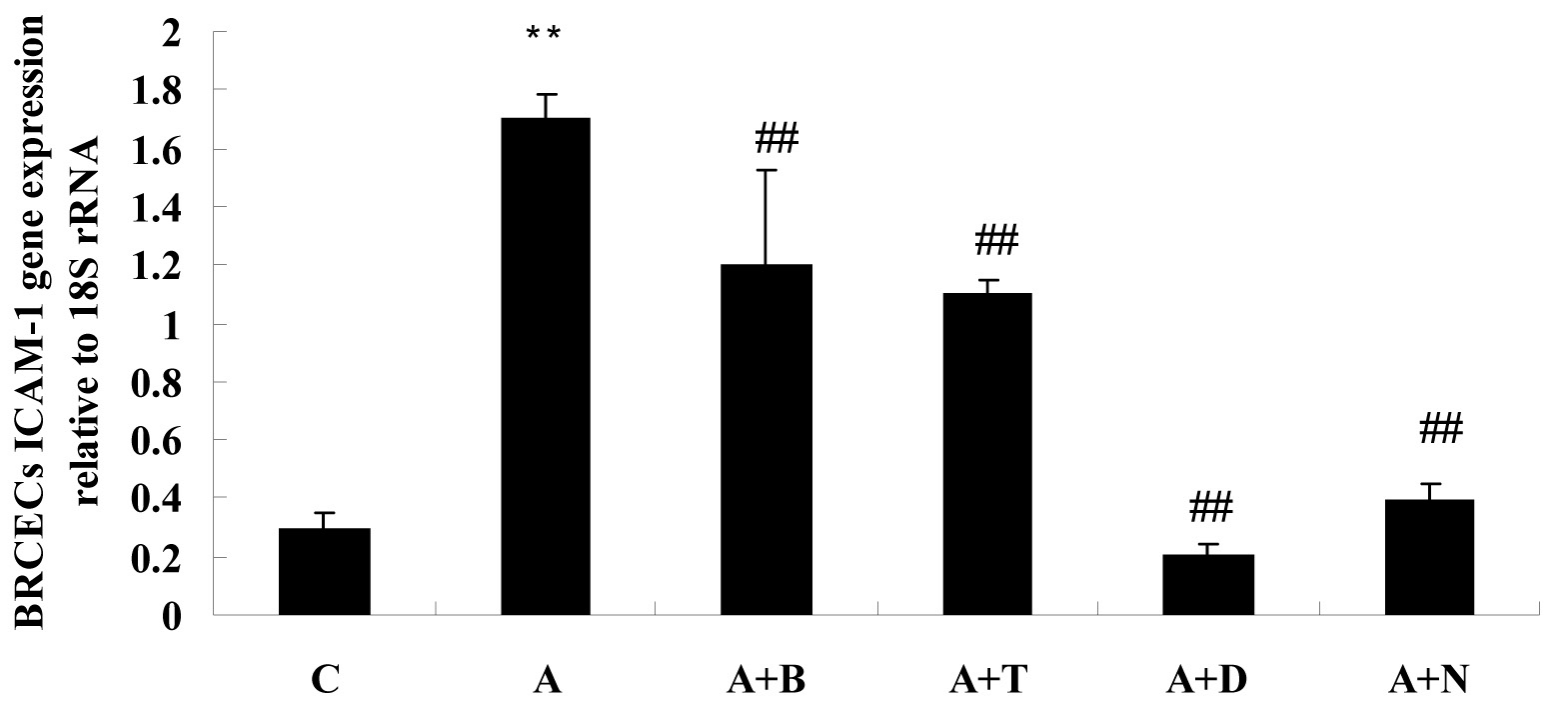
The statistical analysis results of bovine retinal capillary endothelial cells (BRCECs) intercellular adhesion molecule-1 (ICAM-1) gene expression relative to 18S rRNA for 24 h. Asymmetric dimethylarginine (ADMA) markedly upregulated ICAM-1 mRNA expression in BRCECs for 24 h. Benazepril, telmisartan, diphenyliodonium (DPI), or N-acetyl-l-cysteine (NAC) downregulated ICAM-1 mRNA expression in BRCECs exposed to ADMA. In group C, BRCECs were cultured in endothelial cell medium (ECM) with 0.4% fetal bovine serum (FBS). In group A, BRCECs were cultured in ECM with 0.4% FBS plus ADMA (100 μM). “A+B” represents same as for group A and 10 μM benazepril. In group “A+T,” BRCECs were cultured in the same media as group A and 10 μM telmisartan. In group “A+D,” BRCECs were cultured in the same media as group A and 10 μM DPI. In group “A+N,” BRCECs were cultured in the same media as group A and 10 mM NAC (mean±SD, n=3). **p<0.01 versus group C, ##p<0.01 versus group A.

ICAM-1 mRNA expression in BRCECs was markedly upregulated following exposure to ADMA (100 μM) for 24 h (p<0.01). Benazepril (10 μM), telmisartan (10 μM), DPI (10 μM), or NAC (10 mM) downregulated ICAM-1 mRNA expression in BRCECs exposed to ADMA (100 μM; p<0.01; Figure 6).

**Figure 7:**
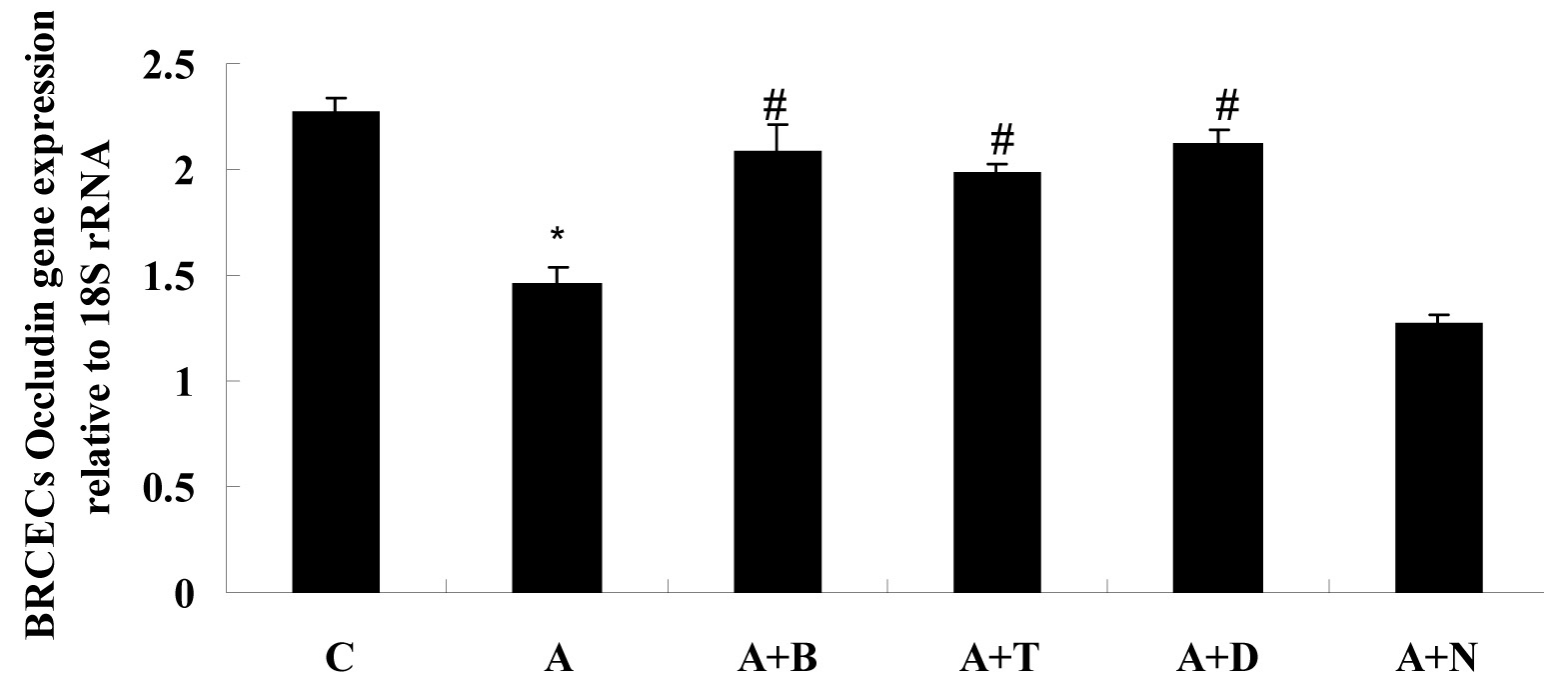
Results of statistical analysis of occludin gene expression relative to 18S rRNA in bovine retinal capillary endothelial cells (BRCECs) for 24 h. ADMA downregulated occludin mRNA expression in BRCECs for 24 h. Benazepril, telmisartan, or diphenyliodonium (DPI) upregulated occludin mRNA level. However, N-acetyl-l-cysteine (NAC) had no effect on occludin mRNA level. In group C, BRCECs were cultured in endothelial cell medium (ECM) with 0.4% fetal bovine serum (FBS). In group A, BRCECs were cultured in ECM with 0.4% FBS plus ADMA (100 μM). In group “A+B,” BRCECs were cultured in the same media as group A and 10 μM benazepril. In “A+T,” BRCECs were cultured in the same media as group A and 10 μM telmisartan. In group “A+D,” BRCECs were cultured in the same media as group A and 10 μM DPI. In group “A+N,” BRCECs were cultured in the same media as group A and 10 mM NAC (mean±SD, n=3). *p<0.05 versus group C, #p<0.05 versus group A.

Occludin mRNA expression in BRCECs was downregulated when cells were exposed to ADMA (100 μM) for 24 h (p<0.05). Treatment with benazepril (10 μM), telmisartan (10 μM), or DPI (10 μM) upregulated the occludin mRNA level in BRCECs exposed to ADMA (p<0.05). However, NAC (10 mM) had no effect on the occludin mRNA level (p>0.05; Figure 7).

## Discussion

Kakimoto and Akazawa [19] first isolated and described ADMA from human urine in 1970. Since their initial observation, ADMA has been shown to represent a novel risk factor for the development of endothelial dysfunction. Oxidative stress has been shown to increase the activity of arginine-methylating and ADMA-degrading enzymes, leading to increased ADMA concentrations; moreover, high ADMA levels further contribute to the vascular oxidative stress burden in a positive feedback fashion [20,21]. As reported previously, BRCECs incubated in the presence of high glucose concentrations showed elevated ROS production, PRMT-1 expression, reduced DDAH activity, and DDAH II expression, and increased the accumulation of ADMA in a conditioned medium [5]. In the present study, we found that ADMA increased intracellular ROS generation in BRCECs. Thus, we propose that ADMA is not only a marker, but also a producer of oxidative stress, under high-glucose conditions.

There have been many recent studies regarding the effects of different therapeutic interventions on ADMA plasma concentrations. ACEI and ARB have been shown to reduce the levels of ADMA and improve endothelial dysfunction in human essential hypertension and diabetes mellitus [22-24]. Although the mechanisms of the beneficial effects of ACEI and ARB remain obscure, ADMA is known to upregulate several components of microvascular RAS, leading to increased production of angiotensin II, which then activates NADPH oxidase and increases ROS production [25]. ADMA improves the p38 mitogen-activated protein kinase activity in human coronary artery endothelial cells, which may provide a link between ADMA and RAS, because ACE protein expression has been shown to be regulated by p38 mitogen-activated protein kinase [26,27]. In the present study, the NADPH oxidase inhibitor DPI or the free-radical scavenger NAC decreased intracellular ROS generation in BRCECs incubated with ADMA. Benazepril or telmisartan had effects similar to those from DPI and NAC, indicating that they may also exert their effects through the oxidase pathway.

It has been reported that ADMA regulates endothelial permeability and endothelial barrier function. Several possible mechanisms have been proposed to explain the effects of ADMA on endothelial barrier function. A previous study indicated that ADMA increased pulmonary endothelial permeability both in vitro and in vivo, and that this effect was mediated by nitric oxide, acting via protein kinase G and independent of ROS formation [16]. Others have demonstrated that ADMA compromises the integrity of the glomerular filtration barrier by altering the bioavailability of nitric oxide and superoxide, and that nitric oxide (NO)-independent activation of soluble guanylyl cyclase preserves the integrity of this barrier under conditions of NO depletion [28]. ADMA markedly downregulated connexin43 expression and damaged gap junction function in human umbilical vein endothelial cells by increasing the production of intracellular ROS and inducing phosphorylation of p38 MAPK [29]. However, to date, the role of ADMA in the BRB has not been studied.

The BRB, an important ocular barrier, consists of two components: the inner and outer BRB. The inner BRB is formed by retinal microvascular endothelial cells with tight junctions between them. The normal inner BRB is determined by the homeostasis of retinal microvessels, and plays a critical role in normal visual function. Enhanced retinal vascular permeability has been shown to aggravate microvascular endothelial cell damage and capillary nonperfusion [30]. Several studies linked inflammation to vascular leakage in diabetic retinopathy. Leukocytes adhere to the retinal vascular endothelium early in diabetic retinopathy, the onset of which occurs before the development of any clinical pathology [14]. Further, leukocyte adhesion coincides with the development of BRB breakdown and capillary nonperfusion [14]. The expression of ICAM-1 is upregulated in DR, and the specific inhibition of ICAM-1 prevents diabetic retinal leukocyte adhesion and BRB breakdown [14,31,32].

In this study, ADMA significantly increased BRCEC permeability within 1 h and was strongly involved in reducing the levels of the tight-junction protein occludin and upregulation of ICAM-1 expression in BRCECs. ACEI, ARB, or antioxidants significantly downregulated cell permeability increased by ADMA at 30 min, 45 min, and 1 h. Furthermore, we observed that benazepril, telmisartan, and the antioxidant DPI reversed the effects of ADMA on occludin and ICAM-1 expression. Based on our findings, we hypothesize that the mechanism of ADMA damage to the BRB is partly mediated by ROS and/or RAS pathways.

In summary, ADMA can inhibit BRCEC proliferation, increase intracellular ROS generation, increase cell permeability and in BRCECs, can reduce levels of the tight-junction protein occludin and increase ICAM-1 expression. Further studies are required to determine the precise mechanisms underlying the effects of ADMA in diabetes-induced BRB breakdown and the roles of ACEI, ARB, and antioxidants in the control of retinal endothelial barrier function under diabetic conditions.
